# Antinociceptive Activity of the Skin Secretion of *Phyllomedusa rohdei* (Amphibia, Anura)

**DOI:** 10.3390/toxins12090589

**Published:** 2020-09-11

**Authors:** Elena Lucia Anna Malpezzi-Marinho, Cristiane Isabel Silva Zanoni, Graziella Rigueira Molska, Camila Paraventi, Raphael Wuo-Silva, Laís Fernanda Berro, Carlos Amilcar Parada, Eduardo Koji Tamura, Eduardo Ary Villela Marinho

**Affiliations:** 1 Biological Sciences Department, Universidade Estadual de Santa Cruz, Ilhéus, Bahia 45662-000, Brazil; 2 Health Area, Universidade Braz Cubas, Mogi das Cruzes, São Paulo 08773-380, Brazil; cristianeisabelsilva@yahoo.com.br (C.I.S.Z.); grazi.molska@gmail.com (G.R.M.); camilaparaventi@gmail.com (C.P.); raphawuo@gmail.com (R.W.-S.); 3 Department of Psychiatry and Human Behavior, University of Mississippi Medical Center, Jackson, MS 39216, USA; berro.lf@gmail.com; 4 Institute of Biology, Universidade Estadual de Campinas, Campinas, São Paulo 13083-970, Brazil; carlosamilcarp@gmail.com; 5 Health Sciences Department, Universidade Estadual de Santa Cruz, Ilhéus 45662-000, Bahia, Brazil; ektamura@yahoo.com.br (E.K.T.); eavmarinho@uesc.br (E.A.V.M.)

**Keywords:** amphibians, antinociceptive activity, pain, *Phyllomedusa rohdei*, skin secretion, frog

## Abstract

Pain is a distressful experience that can have a major impact on an individual’s quality of life. The need for new and better analgesics has been further intensified in light of the current opioid epidemic. Substances obtained from amphibians have been shown to contain bioactive peptides that exert analgesic effects. The genus *Phyllomedusa* represents an important source of peptides and bioactive components. The aim of this study was to investigate the antinociceptive effects of the skin secretion of *Phyllomedusa rohdei* in rodent models of pain. The crude skin extract of *P. rohdei* was tested in different pain models: acetic acid-induced writhing test (mice), formalin test (rats), Von Frey electronic test for hypernociception induced by PGE_2_ (rats), and hot plate test (mice). Motor-impairing effects were tested using the rota-rod test. The results showed that the skin extract of *P. rohdei* exerted antinociceptive effects in all pain models tested. Particularly, the highest dose tested of the skin extract decreased acetic acid-induced writhing by 93%, completely blocked formalin-induced nociception both during the acute and inflammatory phases of the test, PGE_2_-induced hypernociception by 73% and increased latency to paw withdrawal in the hot plate test by 300%. The effects observed in the hot plate test were reversed by pretreatment with selective µ and κ, but not δ, opioid receptor antagonists, indicating a mechanism of action dependent on µ and κ opioid receptors. The results were not influenced by sedative effects. Further studies remain necessary to reveal the specific compounds involved in the antinociceptive effects of *P. rohdei* skin extract as a new therapeutic tool in pain management.

## 1. Introduction

Pain is a major global public health problem. According to the World Health Organization (WHO), one in every five adults frequently experience pain, and one in every ten adults, or 30% of the world population, is diagnosed with chronic pain each year [1,2]. Visceral pain is one of the most common reasons for emergency department visits, and is frequently associated with different types of chronic pain, especially chest and abdominal pain [3]. Because nearly 25% of the population reports having suffered from visceral pain at least once in their lives, health system costs associated with visceral pain are extremely high [3]. Of importance, inflammation is one of the main features associated with chronic pain. Pain and inflammation are regulated by a bidirectional interaction between nociceptor sensory neurons and immune cells, which contributes to pathology in chronic inflammatory diseases such as rheumatoid arthritis, psoriasis, asthmatic lung disease, and colitis [4]. The available therapeutic options for chronic pain, including opioids, are associated with a high number of adverse reactions, tolerance and dependence [5]. Therefore, particularly in light of the current opioid epidemic, new research on treatment alternatives for chronic pain remains necessary.

Venoms/poisons obtained from amphibians have been extensively investigated as new treatments for a variety of medical conditions. Frog skin is a source of many bioactive peptides with a wide range of medical properties, and research has investigated their effects as anti-cancer, anti-viral, immunomodulatory, and anti-diabetic agents [6]. Among the medical uses proposed for these substances, opioid peptides produced by amphibians have also become important targets for biomedical pain research. Opioid peptides identified from the skin of frogs, such as dermorphin and deltorphins, are very similar, and sometimes identical to mammalian gastrointestinal hormones and neurotransmitters [7]. Dermorphin is a potent selective agonist of µ opioid receptors, while deltorphins are potent selective agonists of δ opioid receptor [7,8], suggesting that their effects on pain might be mediated by action at opioid receptors. Both are about one hundred times more potent than morphine [9]. In addition, other types of deltorphin have been proposed to act on Aδ and C fibers, evoking responses from nociceptive neurons of the surface and under the dorsal horn [10].

Because of the pharmacological effects of opioid peptides obtained from frog skin, the cutaneous secretion of some species of frogs has been used for decades as adjuvant therapy in pain management. The Brazilian tribes Mayorama and Maraúbo, and the Peruvian tribes Amahuaca and Matses, use the skin secretion of *Phyllomedusa bicolor*, a species of frog from the Phyllomedusinae subfamily, in rituals, shaving the skin with a piece of bamboo to remove the secretion, which is then dried. The secretion from *P. bicolor*, also known as frog “*kambô*”, is used in folk medicine for pain management in conditions such as premenstrual syndrome and tendinitis. Studies in rodents have further shown that the skin extract of *P. bicolor* has antinociceptive effects in rats that are mediated by opioid receptors [7]. Peptides previously isolated from the skin extract of *P. bicolor* include the opioid peptides dermorphins, dermenkephalins and deltorphins, as well as adenoregulin, vasoactive peptides and antimicrobial agents (dermaseptins) [8,11,12,13]. Because its effectiveness has never been scientifically and systematically proven in humans, disclosing the use of the cutaneous secretion from *P. bicolor* has been prohibited by health surveillance agencies [14]. 

In addition to *P. bicolor*, other species from the Phyllomedusinae subfamily have also been shown to produce bioactive peptides that could be useful for the treatment of acute and chronic pain. *Phyllomedusa rohdei* is one of these species, and from its skin secretion many peptides have been isolated, including filoquinines, fililitorines, sauvagines, triptofilin, rohdei-litorin and dermorphin and its analog [Hyp6]-dermorphin [15,16,17,18]. Although the skin extract of *P. rohdei* is rich in potentially analgesic agents, the antinociceptive activity of this compound has remained to be investigated. Therefore, the aim of the present study was to investigate the antinociceptive effect of the skin secretion of *P. rohdei* in rodent pain models, as well as the opioid mechanisms underlying these effects.

## 2. Results

### 2.1. Writhing Test

**Figure 1:**
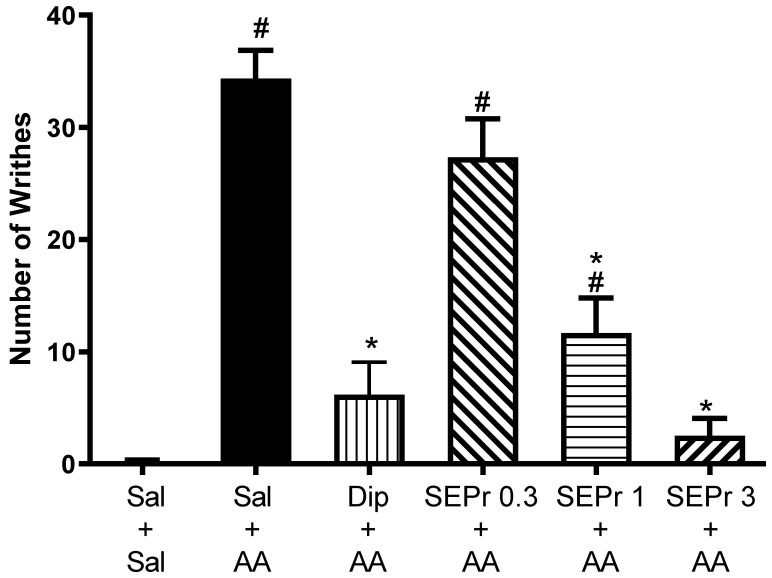
Effects of the skin extract of *Phyllomedusa rohdei* (SEPr) on the writhing test induced by an intraperitoneal injection of acetic acid (AA) in mice. Animals were pre-treated with saline (Sal), dipyrone (Dip, 80 mg/kg) or SEPr (0.3, 1.0 and 3.0 mg/kg) 30 min before saline or AA treatment. Values are presented as mean ± SEM of the number of writhes observed during the 15-min test. # *p* < 0.05 compared to the Saline-Saline group. * *p* < 0.05 compared to the Saline + AA group. One-way ANOVA followed by Bonferroni test, *n* = 6/group.

Figure 1 illustrates the effects of intraperitoneal administration of saline, dipyrone or different doses of a skin extract of *Phyllomedusa rohdei* (SEPr) on acetic acid-induced writhing behavior in mice. Dipyrone (metamizole) was used as a positive control in Experiments 1 and 2, because of its established analgesic effects [19]. 

One-way ANOVA demonstrated a significant difference between groups (F(5,30) = 30.31, *p* < 0.0001). Dipyrone significantly decreased acetic acid-induced writhing. SEPr dose-dependently induced antinociceptive effects. Animals treated with the lowest dose (0.3 mg/kg) of SEPr extract showed no significant differences compared to the control acetic acid group. In contrast, groups treated with 1.0 or 3.0 mg/kg SEPr showed a significant reduction in the number of writhes induced by acetic acid. Of note, 3.0 mg/kg SEPr had a more prominent antinociceptive effect than 1.0 mg/kg SEPr, reducing the number of writhes by 93% (compared to a 66% decrease induced by 1.0 mg/kg SEPr).

### 2.2. Formalin Test

**Figure 2:**
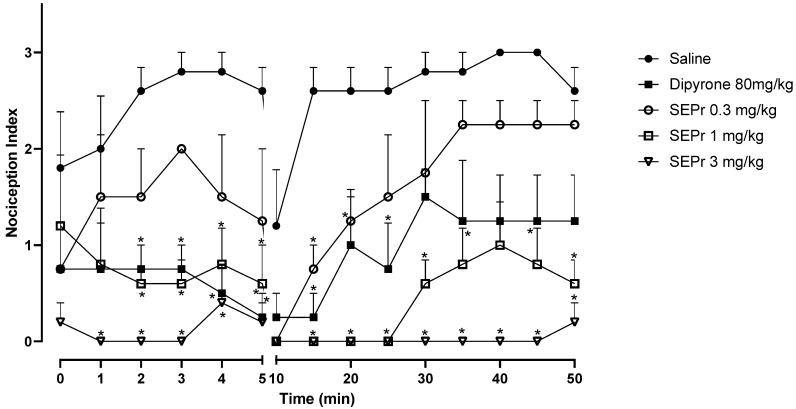
Antinociceptive effects of the skin extract of *Phyllomedusa rohdei* (SEPr) during the acute (0–5 min) and inflammatory (15–50) phases of the formalin test in rats. Animals were pre-treated with saline, dipyrone (80 mg/kg) or SEPr (0.3, 1.0 and 3.0 mg/kg) 30 min before nociception induced by formalin (20 mL, 0.2%). Values are presented as mean ± SEM. * *p* < 0.05 compared to the Saline group. Two-way ANOVA followed by Bonferroni test, *n* = 6/group.

Figure 2 illustrates the effects of intraperitoneal administration of saline, dipyrone or different doses of a skin extract of *Phyllomedusa rohdei* (SEPr) on the acute or inflammatory phases of the formalin test in rats. In rodents, intraplantar injections of formalin produce a biphasic behavioral effect consisting of an acute phase, occurring between 0–5 min after the injection, and a second phase between 20–30 min after injection. A quiescent period separates the two phases, thus the observed drop in the nociception index at 10 min. Studies suggest that the first phase results from the direct stimulation of nociceptors, whereas the second phase involves a period of sensitization during which inflammatory effects occur [20].

During the acute phase (0–5 min), two-way repeated measures ANOVA indicated an effect of treatment (saline, dipyrone or SEPr) (F (4,18) = 12.42, *p* < 0.0001), with no interaction between treatment and time. Dipyrone significantly decreased (*p* < 0.0001) the nociception index during the acute phase. SEPr dose-dependently induced antinociceptive effects. Animals treated with the lowest dose (0.3 mg/kg) of SEPr extract showed no significant differences compared to the control (saline) group. In contrast, groups treated with 1.0 or 3.0 mg/kg SEPr showed a significant reduction (*p* < 0.0001 for both groups) in nociception index. Of note, 3.0 mg/kg SEPr had a faster (already effective at minute 1) and more prominent antinociceptive effect compared to both dipyrone and 1.0 mg/kg SEPr. For example, at minute 3, 3.0 mg/kg SEPr completely blocked nociception, decreasing the nociception index to zero, while dipyrone and 1.0 mg/kg SEPr reduced it by 73% and 78%, respectively.

During the inflammatory phase (minutes 15 to 50) two-way repeated measures ANOVA showed a significant interaction between treatment and time (F (32,144) = 1.939, *p* < 0.01). While both dipyrone and the dose of 0.3 mg/kg SEPr induced analgesic effects that only lasted between minutes 10 and 30, the doses of 1.0 mg/kg SEPr and 3.0 mg/kg SEPr exerted more prominent and longer-lasting antinociceptive effects that lasted until the end of the test at 50 min. Of note, while 1.0 mg/kg SEPr only abolished nociception until minute 25, 3.0 mg/kg SEPr completely abolished formalin-induced nociception until minute 45, with a minimum increase in the nociception index at minute 50.

### 2.3. Hypernociception Test

**Figure 3:**
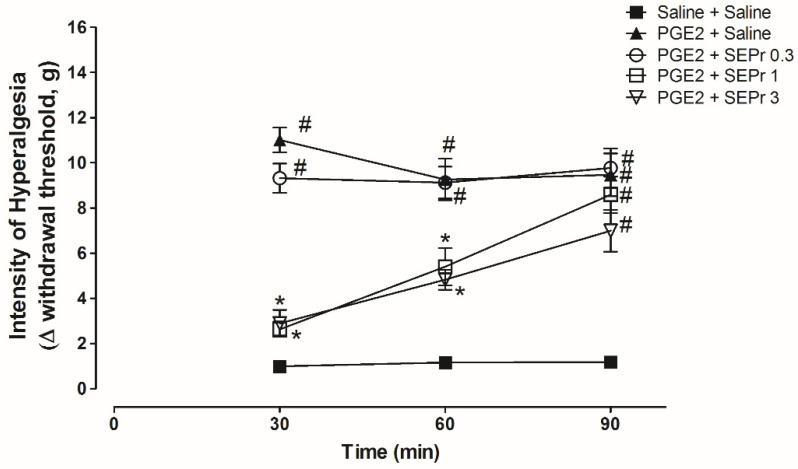
Effects of the skin extract of *Phyllomedusa rohdei* (SEPr) on the mechanic hypernociception induced by intraplantar injection of PGE_2_. Animals were pre-treated with saline or PGE_2_ (100 ng/paw) 150 min before receiving saline or SEPr (0.3, 1.0 and 3.0 mg/kg) and the nociceptive threshold was evaluated by crescent pressure in the rat paw (electronic von Frey) at 30, 60 and 90 min. Values are presented as mean ±SEM. ^#^ *p* < 0.05 compared to the Saline-Saline group. * *p* < 0.05 compared to the PGE2+Saline group. Three-way ANOVA followed by Tukey *post hoc* test, *n* = 5/group).

Figure 3 illustrates the effects of intraperitoneal administration of saline or different doses of a skin extract of *Phyllomedusa rohdei* (SEPr) on hypernociception induced by the administration of prostaglandin E_2_ (PGE_2_) in the paw of rats and measured using Electronic von Frey. 

Three-way repeated measures ANOVA showed a significant effect of time, treatment and their interaction (F(8,40) = 4.45; *p* = 0.0006). As expected, PGE_2_ treatment (PGE_2_ + saline group) induced a significant increase in the withdrawal threshold during the von Frey electronic test for hypernociception compared to the saline control group (saline + saline group) from minutes 30 to 90 (Figure 3). Pretreatment with 1.0 and 3.0 mg/kg SEPr, but not 0.3 mg/kg SEPr, induced a significant and similar antinociceptive effect compared to the PGE_2_ control group at minutes 30 and 60.

### 2.4. Hot Plate Test

**Figure 4:**
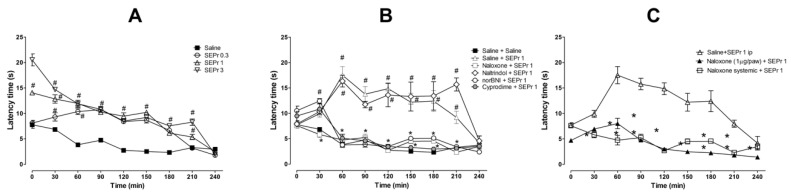
Effects of the skin extract of *Phyllomedusa rohdei* (SEPr) in the hot plate test and the role of opioid receptors in SEPr-induced antinociception. (**A**) Animals were pre-treated with saline or SEPr (0.3, 1.0 and 3.0 mg/kg) 30 min before evaluation of thermal latency for 240 min. (**B**) In a subsequent experiment, animals were pre-treated with opioid antagonists (naloxone 1.0 mg/kg; naltrindole 1.0 mg/kg; norBNI 5.0 mg/kg; cyprodime 8.0 mg/kg) 40 min before saline or SEPr injections and subsequently evaluated in the hot plate test (doses were determined based on previous studies, see Methods section). (**C**) Effects of systemically (1.0 mg/kg) and locally (1.0 µg/paw) administered naloxone in the antinociceptive effects of SEPr (1.0 mg/kg) in the hot plate test. Values are presented as mean ±SEM. ^#^ *p* < 0.05 compared to Saline group (or Saline+saline group). * *p* < 0.05 compared to Saline+SEPr 1 group (or Saline+SEPr 1 ip group). Two- or three-way ANOVA followed by Bonferroni test, *n* = 5/group.

Figure 4 illustrates the effects of intraperitoneal administration of saline or different doses of a skin extract of *Phyllomedusa rohdei* (SEPr) in the hot plate test. The effects of pretreatments with saline or the non-selective opioid antagonist naloxone, the δ receptor selective antagonist naltrindole, the κ receptor selective antagonist norBNI or the µ receptor selective antagonist cyprodime were also investigated on the antinociceptive effects of 1.0 mg/kg SEPr. Finally, the effects of systemic administration of naloxone were compared with the effects of local administration of naloxone in the paw.

For the experiment investigating the effects of SEPr on thermal nociception (Figure 4A), two-way ANOVA with repeated measures showed a significant interaction between treatment and time (F(24,96) = 20.60; *p* < 0.0001). Pretreatment with SEPr at all doses (0.3, 1.0 and 3.0 mg/kg) significantly increased latency to response to thermal stimulus compared with the saline control group. At a dose of 0.3 mg/kg, SEPr significantly increased latency to paw withdrawal from minutes 30 to 180, while pretreatment with 1.0 and 3.0 mg/kg SEPr increased latency throughout the majority of the experiment (minutes 0 to 210) compared to the saline control group. Among the SEPr-treated groups, the SEPr 0.3 group showed lower latency at times 0 and 30 compared to SEPr 1 and SEPr 3 groups, while the SEPr 1 group showed lower latency at times 0 and 210 compared to the SEPr 3 group. Thus, within increasing dose, there was not only a more pronounced effect, but also an increase in the duration of the effect.

To test whether the effects of SEPr in the hot plate test were mediated by opioid receptors, selective and non-selective opioid antagonists were administered prior to administration of SEPr at a dose of 1.0 mg/kg (Figure 4B). Two-way repeated measures ANOVA indicated a significant interaction between treatment and time (F(40,160) = 10.08; *p* < 0.0001). Pretreatment with the non-selective opioid antagonist naloxone, the µ receptor selective antagonist cyprodime and the κ receptor selective antagonist norBNI blocked SEPr-induced antinociception in the hot plate test, decreasing latency to paw withdrawal to control levels. Pretreatment with the δ receptor selective antagonist naltrindole, on the other hand, did not affect SEPr-induced antinociception.

To evaluate if SEPr-induced antinociception was mediated by central vs. peripheral neurons, the non-selective opioid antagonist naloxone was administered systemically or locally in the paw of the animals prior to systemic administration of 1 mg/kg SEPr (Figure 4C). Two-way repeated measures ANOVA showed a significant interaction effect between treatment and time (F(16,64) = 9.44; *p* < 0.0001). Bonferroni post-hoc test showed that pretreatment with naloxone blocked SEPr-induced antinociception in the hot plate test regardless of the administration route (systemic vs. peripheral).

### 2.5. Rota Rod Test

**Figure 5:**
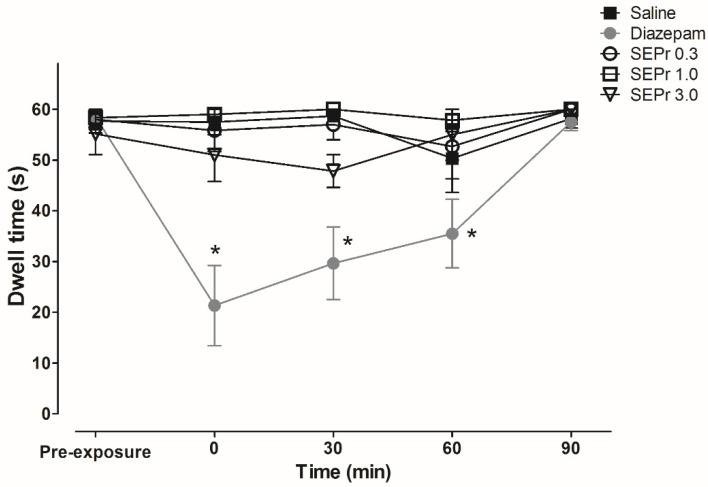
Effects of the skin extract of *Phyllomedusa rohdei* (SEPr) on motor coordination in mice. Animals were treated with Saline, Diazepam (2.0 mg/kg) or SEPr (0.3, 1.0 or 3.0 mg/kg). Data were collected during 60-sec rota-rod sessions the day before drug tests (pre-exposure) and at minutes 0, 30, 60 and 90 after treatments. Values are presented as mean ± SEM. * *p* < 0.05 compared to the Saline group. Two-way ANOVA followed by Bonferroni test, *n* = 6/group.

Figure 5 illustrates the effects of an intraperitoneal administration of saline or different doses of a skin extract of *Phyllomedusa rohdei* (SEPr) on motor coordination in the rota rod test in mice, in comparison with the sedative drug Diazepam. 

Two-way ANOVA indicated a significant interaction between treatment and time (F(16,125) = 3.46; *p* < 0.0001). No significant differences were observed between groups in the pre-exposure test. During the drug tests, 2.0 mg/kg Diazepam induced a significant reduction in length of stay in the apparatus from times 0 to 60. Treatment with SEPr did not affect dwell time at any of the doses tested.

## 3. Discussion

In the present study we described the antinociceptive effects of the crude skin extract of *Phyllomedusa rohdei* (SEPr) in rodent pain models. SEPr dose-dependently blocked nociception induced by systemic acetic acid in mice, local (paw) injection of formalin in rats, local (paw) PGE_2_ injection in rats and heat during the hot plate test in mice. Our results also showed that the antinociceptive effects observed in the hot plate test were reversed by pretreatment with selective µ and κ, but not δ, opioid receptor antagonists, indicating a mechanism of action dependent on µ and κ opioid receptors. Importantly, the results were not influenced by sedative effects, as shown by a lack of effect of SEPr on locomotor activity observed in the rota-rod test. 

In rodents, intraplantar injections of formalin produce a biphasic behavioral effect consisting of an acute phase, and a chronic, inflammatory phase, with studies suggesting that the first phase results from the direct stimulation of nociceptors, whereas the second phase involves a period of sensitization during which inflammatory effects occur [20]. In our study, the effects of SEPr in the formalin test showed that the extract acts by inhibiting both the acute and inflammatory phases of formalin-induced nociception. In contrast, previous studies have shown that the crude skin extract of animals from the Phyllomedusinae subfamily alone, such as the *Phyllomedusa hypochondrialis,* injected directly in the paw induce nociception and increase inflammatory parameters, such as leukocyte migration and vascular permeability [21,22]. Locomotor activity was also compromised by systemic administration of *P. hypochondrialis* skin extract [22], corroborating previous work from Daly et al. [11] with crude skin extract from *P. bicolor.* In the present study, however, *P. rohdei* extract did not affect baseline locomotor activity and, contrary to that observed with the skin extract from other species from the Phyllomedusinae subfamily, SEPr had antinociceptive activity in all pain models evaluated in our study. 

Considering the wide array of bioactive peptides present in their skin, Phyllomedusinae frogs have contributed to the study of skin peptides with potential biological actions, such as antimicrobial, antinociceptive, hormonal and neural activities [23,24]. The skin secretion of animals from the genus *Phyllomedusa* contains several bioactive peptides, including dermorphins, phylloseptins, dermaseptins, medusins and phyllokinins, and more than 20 different peptides have already been described in the skin secretion of the *P. rohdei* species [23]. Thus, the analgesic effects of *P. rohdei* could be mediated by opioidergic activity from the peptides present in the animals’ skin, and some of the peptides isolated from amphibians seem to exert their effects through interaction with opioid receptors [23]. In fact, in the present study, the antinociceptive effects of SEPr were reversed by pretreatment with opioid receptor antagonists. It is important to note, however, that we did not characterize the bioactive peptides from our specific sample of SEPr, which is an important limitation of the present study. Therefore, further studies are warranted to identify which specific bioactive peptides are responsible for the antinociceptive effects of SEPr. Although we were unable to perform such characterization, in the present study we sought to determine whether opioid mechanisms were involved in SEPr-induced antinociception, and which opioid receptors were responsible for these effects.

In the present work, both the selective antagonist of µ opioid receptors, cyprodime, and the selective antagonist of κ opioid receptors, norBNI, reversed the antinociceptive effects of SEPr, indicating a mechanism of action dependent on both µ and κ opioid receptors. Corroborating the present findings, dermorphin-like peptides isolated from *P. bicolor* also induced analgesic effects in a rodent tail-flick nociception model, an effect that was inhibited by pretreatment with the non-selective opioid receptor antagonist Naloxone [7]. In the same work, Negri et al. [7] showed that the effects of the isolated peptides were associated with activation of µ opioid receptors as suggested by binding assays. 

A major problem associated with the use of opioids for pain management is that most opioids that act through µ opioid receptors induce marked side effects at high doses and have abuse liability, including a great number of emergency room visits involving prescription opioid misuse [25]. Recent evidence suggests that substances acting more selectively at peripheral κ opioid receptors also produce analgesia with reduced central nervous system side effects [25,26]. Agonists of κ opioid receptors not only act as analgesics, but also as anti-inflammatory agents [27]. Thus, by acting at both µ and k opioid receptors, SEPr would have a broader effect, leading to analgesia at doses that do not induce major side effects. In fact, the present findings indicate that SEPr did not affect baseline locomotor activity in the rota rod test. In addition, the antinociceptive effects of SEPr were reversed by local (intraplantar injection) antagonism of opioid receptors with the non-selective antagonist Naloxone. These findings suggest that the analgesic effects of SEPr can be achieved through activation of peripheral opioid receptors, not necessarily involving a central nervous system pathway. In agreement, Albert-Vartanian et al. [25] previously suggested that treatment with opioids in a peripherally restricted manner could exert antinociceptive effects with a decrease in the emergence of unwanted adverse effects and abuse liability compared to the use of opioids acting directly in the central nervous system [25]. 

In conclusion, our findings show that SEPr can exert antinociceptive effects with lack of motor side effects and without depending on central mechanisms. The exact mechanisms underlying the antinociceptive effects of SEPr remains unknown, and further studies with the isolated compounds of SEPr are necessary to evaluate the efficacy and safety of the peptides present in the extract. We emphasize, however, that attention is needed regarding the ecological impact of conducting studies with endemic frog species and that strategies are necessary in order to assure the preservation and conservation of endangered species and their habitats. 

## 4. Conclusions

The skin extract of *P. rohdei* exerted antinociceptive effects in all pain models tested in the present study. The antinociceptive effects observed in the hot plate test were reversed by pretreatment with selective µ and κ, but not δ, opioid receptor antagonists, indicating a mechanism of action dependent on µ and κ opioid receptors. The results were not influenced by sedative effects, as shown by a lack of effect of the skin extract on locomotor activity observed in the rota rod test.

## 5. Materials and Methods

### 5.1. Collection of Anurans

Four specimens of *Phyllomedusa rohdei* (Brazilian Institute of the Environment and Renewable Natural Resources—IBAMA license No 16236-1), were collected in the city of Biritiba Mirim, in São Paulo State, Brazil, located in the Mata Atlântica region (Latitude: 23° 34′ 26″ S, Longitude: 46° 2′ 19″ W).

### 5.2. Skin Extract 

Animals were euthanized by decapitation, their skin removed and maintained in methanol (100 mL/skin) for 30 days under room temperature. At the end of the 30-day period, the methanol was filtered, removed by concentration on a rotative evaporator and subsequently freeze-dried, and named Skin Extract of *Phyllomedusa rohdei* (SEPr). 

### 5.3. Animals

Male 3-month-old Swiss mice (25–35 g) and Wistar rats (200–250 g) were obtained from the vivarium of the University of São Paulo/Ribeirão Preto or from Braz Cubas University. Animals were housed under a controlled temperature (22 ± 1 °C) in a light/dark cycle of 12 h (lights on at 7:00 a.m.) with free access to food and water. Mice and rats were acclimated to the testing laboratory for at least 1 h before the beginning of testing and used once throughout the experiments. The experiments described in the present study were performed in accordance with the National Institute of Health Guide for the care and use of laboratory animals (8th edition, revised 2011), and animals were maintained in accordance with the Brazilian Law for Procedures for Animal Scientific Use (#11794/2008). All experimental procedures were approved by the Institutional Animal Care and Use Committee of Braz Cubas University (protocol #160/2008). The date of approbation for the ethical committee was 18 June 2008.

### 5.4. Chemicals

Formalin, acetic acid, prostaglandin E_2_, naloxone, naltrindole, norBNI, and cyprodime were acquired from Sigma-Aldrich (St. Louis, MO, USA). Diazepam was acquired from Roche (Basel, Switzerland) and all other chemicals used were of analytical reagent grade.

### 5.5. Experimental Procedures

The experiments described below were conducted either in mice or in rats. The species used for each experiment was mentioned in the description of each of the Experimental Procedures. Rats were used for experiments that required manipulation of the animals’ paws (Formalin test and Hypernociception test), while mice were used for the remaining experiments.

#### 5.5.1. Writhing Test

The writhing test was performed as previously described by Niemegeers et al. [28]. Writhing behavior was induced by intraperitoneal injection of 0.4 mL of acetic acid (0.6%) diluted in distilled water. Five groups of six mice each were pre-treated with intraperitoneal injections of saline (control group), dipyrone (80 mg/kg, positive control group) or SEPr (0.3, 1.0 and 3.0 mg/kg) 30 min before saline or acetic acid administration. Immediately after acetic acid injections, mice were placed in individual glass tanks and the number of writhing behaviors was measured cumulatively over 15 min.

#### 5.5.2. Formalin Test

The formalin test was performed according to the method previously described by Le Bars et al. [20]. Six groups of six rats each were treated with an intraperitoneal injection of saline (control group), dipyrone (80 mg/kg, positive control group) or SEPr (0.3, 1.0 and 3.0 mg/kg) 30 min before the administration of 20 mL of 0.2% formalin (Saline, SEPr 0.3-, SEPr 1.0- and SEPr 3.0 groups) on the plantar surface of the right hind paw. After formalin injection, rats were immediately placed in an open-field arena with a wooden floor of 40 cm in diameter for evaluation of nociceptive behavior. Animal behavior was measured using the following nociceptive score: 0—normal posture; 1—injected paw remaining on the ground but not supporting the animal; 2—injected paw clearly raised; 3—injected paw being licked, nibbled, or shaken. The different parameters were timed every minute during the first 5 min and every 5 min from minutes 5 to 50. The nociception index was determined by the mean of the pain score for each group of rats in each time-point.

#### 5.5.3. Hypernociception Test

The term hypernociception was used to define the decrease in nociceptive withdrawal threshold [29]. Briefly, in a quiet room, rats were placed in acrylic cages (12 × 20 × 17 cm) with wire grid floors 30 min before the beginning of the test. The test consisted of evoking a hind paw flexion reflex with a hand-held force transducer adapted with a 0.7 mm^2^ polypropylene tip (Electronic von Frey; IITC Life Science, Woodland Hills, CA, USA). The investigator was trained to apply the tip perpendicular to the central area of the hind paw with a gradual increase in pressure. The end-point was characterized by the removal of the paw followed by clear flinching movements. After paw withdrawal, the intensity of the pressure was automatically recorded, and the final value for the response was obtained by averaging three measurements. The animals were tested before and after treatments. Five groups of five rats each were treated with an intraperitoneal injection of saline (control group) or SEPr (0.3, 1.0 and 3.0 mg/kg) 150 min after the administration of saline (Saline-Saline group) or prostaglandin E_2_ (PGE_2_) 100 ng/paw; Saline-PGE_2_ and SEPr 0.3-, SEPr 1.0- and SEPr 3.0-PGE_2_ groups in the plantar surface of the right hind paw. Prostaglandins are known to cause hyperalgesia, i.e., to lower the pain threshold to mechanical and chemical stimulation in man and other species. This effect seems to be peculiar to prostaglandins among the several putative inflammatory mediators [30]. PGE_2_ and the dose of 100 ng/paw were chosen based on previous research from our group [31]. Although no positive control group was included in this experiment, previous studies from our group have shown that dipyrone is effective in decreasing PGE_2_-induced hypernociception [32]. After PGE_2_ injection, rats were immediately placed in acrylic cages for evaluation of nociceptive behavior. The mechanical nociceptive threshold was evaluated by crescent pressure in the rat paw (electronic von Frey) 30, 60 and 90 min after SEPr injection. Hypernociception was calculated as the difference between basal and post-treatment mechanical nociceptive thresholds (Δ).

#### 5.5.4. Hot Plate Test

The hot plate test was performed as previously described [33,34]. The thermal stimulation of the hot plate apparatus (10 cm wide glass cylinder on a hot plate—IITC Life Science Inc., Woodland Hills, CA, USA) was maintained at 55 ± 1 °C. Mice were placed over the heated surface and the latency to jump or licking response was measured. Two control latencies at least 10 min apart were determined for each mouse. Animals were removed from the apparatus right after the expression of nociceptive behavior. A latency period of 30 s was defined as complete antinociception (cut-off). Four groups of five mice each were treated with intraperitoneal injections of saline (control group) or SEPr (0.3, 1.0 or 3.0 mg/kg) 30 min before being placed in the apparatus, and the thermal latency was evaluated every 30 min during 240 min. Although no positive control group was included in this experiment, previous studies have shown that dipyrone is effective in decreasing PGE_2_-induced hypernociception [35]. The experiment was then repeated for 1.0 mg/kg SEPr, with pretreatments of saline or the non-selective opioid antagonists naloxone (1.0 mg/kg), the δ receptor selective antagonist naltrindole (1.0 mg/kg), the κ receptor selective antagonist norBNI (5.0 mg/kg) and the µ receptor selective antagonist cyprodime (8.0 mg/kg), injected subcutaneously 40 min before the SEPr extract. The doses of opioid antagonists were based on previous studies from our group or on the literature [36,37]. Comparatively, the peripheral action of the non-selective opioid antagonist naloxone was evaluated by administering it to the animals’ paws (1 µL/paw). 

#### 5.5.5. Rota Rod Test

To rule out possible nonspecific muscle-relaxant or sedative effects of SEPr, mice were tested on the rota rod apparatus, as previously described [33,34]. Twenty-four hours before the test, all animals were tested in the rota rod apparatus and only mice that remained on the bar for two consecutive periods of 60 s were considered for the test on the following day. Five groups of six mice each were treated with an intraperitoneal injection of saline (control group), SEPr (0.3, 1.0 or 3.0 mg/kg), or Diazepam (2.0 mg/kg) for the positive control. Results were expressed as the time that the animals remained on the rota rod in a 60 s session in three different time-points: before treatments (basal) and 30, 60 and 90 min after injections.

### 5.6. Statistical Analysis

Before conducting the statistical analysis, all variables were checked for normality (Shapiro–Wilk test) and homogeneity (Levene’s test), which validated the use of the parametric tests. Data were presented as means and standard error of means (SEM) and analyzed by one- two- or three-way analysis of variance (ANOVA) with repeated measures when appropriate followed by Bonferroni or Tukey post hoc tests. A p value less than 0.05 was considered as a statistically significant difference.

## Abbreviations

SEPr	Skin Extract of Phyllomedusa rohdei
PGE2	Prostaglandin E2
